# Hydatidiform Mole—Between Chromosomal Abnormality, Uniparental Disomy and Monogenic Variants: A Narrative Review

**DOI:** 10.3390/life13122314

**Published:** 2023-12-10

**Authors:** Andreea Florea, Lavinia Caba, Ana-Maria Grigore, Lucian-Mihai Antoci, Mihaela Grigore, Mihaela I. Gramescu, Eusebiu Vlad Gorduza

**Affiliations:** 1 Department of Medical Genetics, Faculty of Medicine, “Grigore T. Popa” University of Medicine and Pharmacy, 700115 Iasi, Romania; andreeaflorea97@gmail.com (A.F.); anagrigore2626@gmail.com (A.-M.G.); lucian-mihai.antoci@email.umfiasi.ro (L.-M.A.); mihaelagramescu@yahoo.ro (M.I.G.); vgord@mail.com (E.V.G.); 2 Department of Obstetrics and Gynecology, “Grigore T. Popa” University of Medicine and Pharmacy, 700115 Iasi, Romania; mihaela.grigore@edr.ro

**Keywords:** hydatidiform mole, uniparental disomy, triploidy, androgenetic, monogenic variant

## Abstract

A hydatidiform mole (HM) or molar pregnancy is the most common benign form of gestational trophoblastic disease characterized by a proliferation of the trophoblastic epithelium and villous edema. Hydatidiform moles are classified into two forms: complete and partial hydatidiform moles. These two types of HM present morphologic, histopathologic and cytogenetic differences. Usually, hydatidiform moles are a unique event, but some women present a recurrent form of complete hydatidiform moles that can be sporadic or familial. The appearance of hydatidiform moles is correlated with some genetic events (like uniparental disomy, triploidy or diandry) specific to meiosis and is the first step of embryo development. The familial forms are determined by variants in some genes, with *NLRP7* and *KHDC3L* being the most important ones. The identification of different types of hydatidiform moles and their subsequent mechanisms is important to calculate the recurrence risk and estimate the method of progression to a malign form. This review synthesizes the heterogeneous mechanisms and their implications in genetic counseling.

## 1. Introduction and Objectives

Gestational trophoblastic disease (GTD) comprises a series of gestational disorders that vary from benign to malignant forms. The benign form of GTD is represented by a hydatidiform mole (HM), which is the most common. The malignant forms of GTD are represented by trophoblastic tumors (with two forms—epithelial trophoblastic and placental site trophoblastic tumors), choriocarcinoma and invasive moles [1].

Moles have been described since antiquity by Hippocrates, but the term mole was introduced in 1752 by William Smellie [2]. The hydatidiform mole, commonly known as a molar pregnancy, is a rare complication in gestation, mainly caused by a genetic error during fertilization or gametogenesis [3,4]. This is characterized by a disordered proliferation of the trophoblastic epithelium and villous edema [5,6].

Morphologic, histopathologic and cytogenetic criteria can identify two types of hydatidiform moles: a complete hydatidiform mole (CHM) and partial hydatidiform mole (PHM) [7]. In the majority of cases with a CHM, the genetic material has paternal origin, being generated by errors of meiosis or fertilization, or during the first steps of embryo development. Usually, a complete hydatidiform mole is a unique event during the reproductive period of women. Although unusual, when recurrence occurs, it is indicative of a genetic predisposition. In partial hydatidiform moles, an imbalance is present between the maternal and paternal genomes with a preponderance of the male genome [2]. The main characteristics of CHMs and PHMs are presented in Table 1.

Epidemiological studies have revealed variable incidence rates of molar pregnancies across different global regions (0.2–9.9 per 1000 pregnancies) [6]. In North America and Europe, the frequency is estimated within the range of 60 to 120 cases per 100,000 pregnancies [8]. However, the incidence is more elevated in other geographic regions, particularly in Asia (especially in Southeast Asia) and Africa, with rates up to ten times higher compared with those of developed countries [2,6]. In developed countries, the incidence of complete hydatidiform moles is approximated at one to three cases per 1000 pregnancies, while those of PHMs hover at about three cases per 1000 pregnancies [2].

In rare situations, a twin pregnancy is affected by a mole. There are two distinct categories of twin molar pregnancies. The most frequent one is a dizygotic twin pregnancy with a coexistence between a CHM in one twin and a normal fetus and placenta in the other twin, which occurs in 1 per 22,000–100,000 pregnancies. In these cases, the placental mass has two components: the normal placenta that serves the normal fetus and the molar placenta [12].

The rate of favorable evolution of such pregnancies is about 40%. The second variant entails an analogous twin configuration, but with a partial hydatidiform mole in one twin, which evolves into a viable fetus in fewer than 25% of instances [13].

The classic complications of hydatidiform moles during pregnancy are spontaneous abortions, intrauterine death, hyperthyroidism and preeclampsia [12]. Whatever the prognosis of hydatidiform moles is worth, the majority of such pregnancies generate a miscarriage or an intrauterine death [3,5].

It is important to differentiate between complete hydatidiform moles, partial hydatidiform moles and non-molar pregnancies for several reasons: the different management in cases of hydatidiform moles and non-molar pregnancies and the risk of recurrence of molar pregnancies in the same individual and in the family is also different [14]. In medical practice, serum β-hCG (human chorionic gonadotropin) and ultrasound are used to identify hydatidiform moles. Histological criteria and genetic analyses are used to discriminate between the two types of HMs and hydropic abortion (HA) [9].

The microscopic appearance is different in complete hydatidiform moles, partial hydatidiform moles and HAs. There are overlaps in histological signs between the two types of moles, but also between molar and non-molar pregnancies. In this last case, differential diagnosis problems may arise when there are pregnancies with abnormal villous morphology or hyperplastic ones, products of mosaic conception or those cases where there is a chimeric conception [9,15].

An proportion of 50% of the partial hydatidiform mole cannot be diagnosed by routine histological examination. This occurs for at least two reasons: there are no specific histological signs and there is a significant inter- and intra-observer variability [16].

Some morphological criteria depend on the gestational age. Thus, ultrasonographic signs of an abnormal pregnancy can lead to therapeutic abortion, but the histopathological examination may not reveal histological signs characteristic of molar pregnancy, because they have not yet appeared [9,15,17].

Sarmadi et al. analyzed various histological criteria belonging to the following category of histological diagnosis criteria: trophoblastic proliferation, villous stroma, villous shape and presence of fetal structures [9]. Using Kappa statistics, the authors concluded that there are morphological criteria with high specificity such as “cistern formation” and “hydropic change” (Kappa value: 0.746 and 0.686, respectively). Kappa values between 0.549 and 0.412 are associated with moderate agreement rates. This category included atypia in trophoblastic cells, trophoblastic-free cell cluster, predominantly syncytiotrophoblastic proliferation, predominantly cytotrophoblastic proliferation, polypoid/lobulated villus, chorionic membrane and nucleated red blood cells. Other histological criteria had a slight agreement rate: a round inclusion (Kappa = 0.174) and an irregular inclusion (Kappa = 0.136). The other criteria used had a fair degree of agreement rate: nuclear debris in the villous stroma, stromal fibrosis (fibrotic chorionic villi), scalloping villus and round villus [9].

Immunohistochemical tests (IHC) are applied for stromal and cytotrophoblastic cells. P57 IHC cannot discriminate between a diploid non-molar hydropic abortion (HA), a partial hydatidiform mole, triploid digynic–monoandric gestations and a trisomy. In all these situations, maternal alleles are present. In this case, in order to increase the detection rate, complementary methods should be applied [17]. The short tandem repeat (STR) profile with the presence of paternal alleles in at least two loci establishes the diagnosis of a CHM [18].

The treatment in gestational trophoblastic disease is different depending on the classification in 1 of the 4 entities: hydatidiform mole, trophoblastic tumor, choriocarcinoma and invasive mole. The standard treatment includes combinations of chemotherapy, dilation and curettage, and hysterectomy. When choosing the treatment option, the woman’s wish to preserve her fertility will also be considered. In all cases, a post-treatment follow-up is necessary to exclude the recurrence of the disease. Management can be challenging in some cases. For example, there is no consensus regarding the application of a prophylactic treatment in cases of a hydatidiform mole with a risk of persistence of the disease or an attitude of following the levels of human chorionic gonadotropin and the application of treatment measures when the criteria of persistence of the disease are met [4].

**Objectives:** The main objectives of this review are to synthesize the heterogeneous mechanisms (chromosomal abnormality, uniparental disomy and monogenic variants) involved in the pathogenesis of the hydatidiform mole and their implications in genetic counselling.

## 2. Materials and Methods

This narrative review centers on the etiology of the hydatidiform mole and its connection to genetic factors. A comprehensive literature search was conducted through the PubMed and Web of Science/Clarivate Analytics databases. The search included articles published in the last 20 years, but we tried to select the most recent ones as research in the genetics field is continuously growing. Only articles available as full-text and in English have been included. The exclusion criteria were conference abstracts, editorials, non-English publications. The electronic search was conducted following different combinations of five keywords: hydatidiform mole, uniparental disomy, triploidy, androgenetic and monogenic variant.

## 3. Results and Discussion

### 3.1. Mechanisms in Hydatidiform Mole

The mechanisms of the hydatidiform mole are different in the complete hydatidiform mole and in the partial hydatidiform mole. In the complete hydatidiform mole, a complete paternal uniparental disomy is implied. In a PHM, a form of polyploidy is present [2].

#### 3.1.1. Genetic Particularities of the Complete Hydatidiform Mole

**Figure 1:**
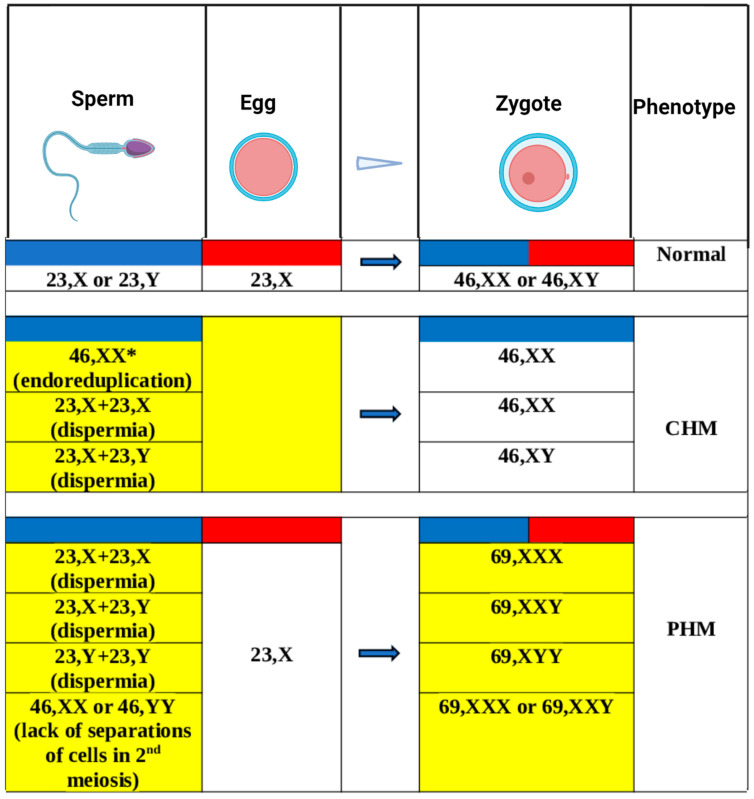
Main mechanisms in the hydatidiform mole: chromosomal abnormality and uniparental disomy. * Result of endoreduplication of the paternal genome; blue—paternal genome; red—maternal genome; yellow—abnormal genetic material quantity/phenomena. Created with Biorender.com (accessed on 19 September 2023).

In the case of a complete hydatidiform mole, the amount of genetic material is normal (with a diploid normal chromosomal formula 46,XX or 46,XY), but the origin of all chromosomes is only paternal. The 46,XX karyotype is identified in 90% of cases [19]. There are two possible mechanisms: endoreduplication and dispermia. In the first case, an enucleated egg is fertilized by a normal sperm, and then the genetic material of paternal origin is duplicated (15% of cases). In the second case, a fertilization error occurs: one enucleate oocyte is fertilized by two genetically normal sperms or by a diploid sperm (resulting from an error of male meiosis—diandry) (Figure 1). Another possible mechanism concerns an error of fertilization—dispermia—with the formation of a triploid embryo, followed by the loss of maternal genetic material [20]. In 15–25% of complete hydatidiform moles, the mechanism is dispermia [14]. Thus, in 80–90% of complete hydatidiform moles, there is a diploid androgenetic genome and, in 10–20%, the genetic contribution is biparental [2,11,21]. In cases with paternal origin, there are some abnormal genetic phenomena called paternal complete uniparental disomy, characterized by the presence of all chromosomal pairs with paternal uniparental origin.

Other genetic changes implied in complete hydatidiform moles are the presence of a maternal mutation that affects imprinting in the offspring. In this situation, the genetic material has a biparental origin, with a normal 46,XX or 46,XY karyotype [20].

Genomic imprinting is an important event in normal embryonic development [22,23]. The phenomenon of parental or genomic imprinting involves epigenetic changes that allow a uniparental allelic expression, either maternal or paternal. The human genome contains about 200 imprinted genes, located on different chromosomes which represent less than 1% of all genes. Some imprinted genes have roles in growth, viability and physiological functions [23]. The *CDKN1C* gene (Cyclin-dependent kinase inhibitor 1C) (alias symbols P57; KIP2), located at 11p15.5, encodes p57^KIP2^, a Cyclin-dependent kinase inhibitor 1C (Cyclin-dependent kinase inhibitor p57). The chromosomal region 11p15.5 is one of the main chromosomal regions characterized by parental imprinting. The *CDKN1C* gene presents a paternal imprinting pattern and thus, normally, only the maternal allele is expressed [22,24]. The immunohistochemistry studies on embryonic tissues show a loss of p57^KIP2^ staining in cytotrophoblast and villous stromal cells in CHMs that is concordant with the absence of this protein, generated by the absence of a maternal allele. On the other hand, P57^KIP2^ immunohistochemistry in PHMs revealed no loss of p57 staining, confirming the presence of an active maternal allele [25].

#### 3.1.2. Chromosomal Abnormality—Triploidy

A partial hydatidiform mole is associated with a polyploid status in the embryo. Usually, there is a triploidy with a paternal extra haploid set of chromosomes. The triploidy is generated in the majority of cases by dispermia, while the rest are produced by a diandry. The most frequent chromosomal formula is 69,XXX karyotype (90% of cases) [19] (Figure 1). In a PHM, both parental genomes are expressed, but this expression is unbalanced with an excess of one of the genomes, maternal or paternal. This imbalance produces anomalies of development that concern the embryo and the placenta. Paternal triploidy is characterized by an abnormal development of the placenta, with the presence of a partial hydatidiform mole in association with relatively minor changes in the embryo (usually microcephaly) [5,19]. Other karyotypes (diploid biparental, triploid digenic, tetraploid triandric) have occasionally been identified [2].

#### 3.1.3. Monogenic Variants and Recurrent Hydatidiform Mole

A recurrent hydatidiform mole (RHM) is a rare form of the complete hydatidiform mole, being discovered in less of 1% of all cases of complete hydatidiform moles. This disorder is characterized by the occurrence of two or more molar pregnancies in the same patient [26]. RHMs can be isolated or familial. The isolated (sporadic) form is generated by a dispermic triploid or androgenetic diploid. In the familial forms, the origin is monogenic, and the embryonic genome is a biparental diploid [20].

Table 2 synthesizes genes involved in RHMs. The most common mutant variants concern the *NLRP7* gene (55% of cases) and the *KHDC3L* gene (5% of cases) [20].

In familial RHMs, the fertilization is normal, but the haploid ovum carries a mutation in either the *NLRP7* or *KHDC3L* gene. Both genes present a monoallelic pattern of expression, produced by parental imprinting. In cases with RHMs, there are mutations in the *NLRP7* or *KHDC3L* genes that produce an inactivation of the maternal allele. Thus, only the paternal allele remains active and its effect on the placenta is similar to that of a male monopaternal diploid genomic expression. Probably, *NLRP7* or *KHDC3L* mutations disrupt defective placental-specific imprinting mechanisms with an unbalanced expression between maternal and paternal alleles. Therefore, we see a biparental recurrent diploid CHM appear [2].

The *NLRP7* gene belongs to the NLR family gene. Members of this gene family are immunoregulatory proteins characterized by the presence of the NACHT nucleotide-binding domain (NBD) and leucine-rich repeats (LRRs). This family contains 25 genes with a role in inflammation and apoptosis [27,30]. *NLRP7* is the first gene whose mutations have been associated with RHMs in over 55% of cases [20]. The *NLRP7* gene is expressed in the oocyte’s stages and the pre-implantation stages of the embryo, but also in the endometrium, placenta and hematopoietic cells. In the embryo, the protein level is different in different stages with a minimum in the blastocyst stage (day 3) and a sudden increase on days 3–5 [31]. Also, this protein has a crucial role in oocyte maturation and in placental development. NLRP7 allows trophoblast proliferation but has a negative regulatory role in trophoblast differentiation. Thus, the abnormal level of NLRP7 expression is associated with excessive trophoblast differentiation [32,33].

**Figure 2:**
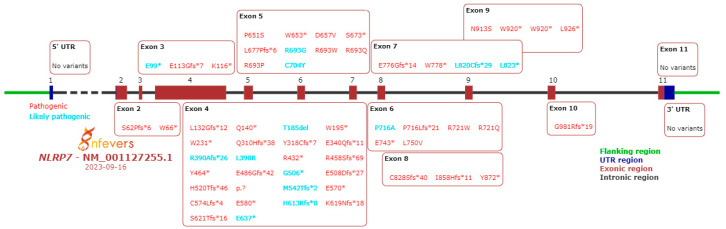
Pathogenic and likely pathogenic variants in *NLRP7* gene exons (“*” name as first published/submitted to Infevers database).

**Figure 3:**
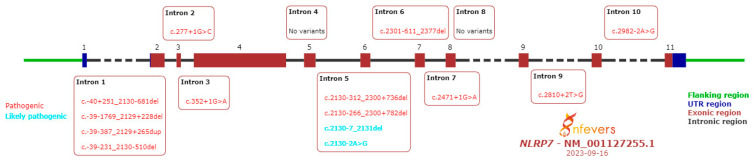
Pathogenic and likely pathogenic variants in *NLRP7* gene introns.

To date, more than 275 variants in the *NLRP7* gene have been identified. They are reported in the INFEVERS database. Figure 2 and Figure 3 summarize the pathogenic and likely pathogenic variants in the *NLRP7* gene [34,35,36,37,38].

Slim et al. analyzed variants in the *NLRP7* gene. Thus, it was observed that there are variants associated with the disease in all exons except exon 11. The most frequent variants are p.Leu750Val (24%), p.Arg693Pro (8.8%) and p.Leu825* (5%). Most variants (73.2%) determined the truncation of the protein and, in 26.7% of cases, they are missense variants. The distribution of these two types of variants is different: protein-truncating variants are dispersed throughout the gene, while missense variants are found especially in the leucine-rich repeat. Protein-truncating variants have a more severe phenotypic effect. Some missense variants are not pathogenic and their presence was associated with normal pregnancy [20]. Some of the mutations of the *NLRP7* gene are characterized by a founder effect. Thus, p.Leu750Val (79% of the alleles) and p.Arg937_Leu938ins54 are specific to Mexicans cases, p.Arg693Trp are frequent in Caucasians and Turks, p.Arg693Trp represents 39% of the mutant alleles in the Indo-Pakistani population, p.Arg693Gln is characteristic of the Chinese population, while p.Glu710Aspfs (32% of the alleles) was found in Egyptians [20,39].

Pathogenic variants in the *NLRP7* gene can induce diploid biparental molar conception, early embryo cleavage abnormalities, non-molar abortions, stillbirths and intrauterine growth retardation [40,41,42,43].

The androgenetic CHMs and the diploid biparental mole determined by *NLRP7* variants have the same phenotype. The difference is that in the second situation, the phenotype is the milder one [20].

The *KHDC3L* gene encodes KH domain-containing protein 3. Transcripts of this gene have been highlighted in hematopoietic cells, all oocytes’ stages and preimplantation embryos that play a role in the development of the ovaries and embryo [31,44]. Like NLRP7, KH domain-containing protein 3 is a constituent of the oocyte cytoskeleton located mainly in the cortical region [45]. Mutations in the *KHDC3L* gene cause an abnormal development of oocytes and molar pregnancies after fertilization [31,44].

The *PADI6* gene is another maternal-effect gene. It encodes an enzyme called protein-arginine deiminase type-6 involved in citrullination. Citrullination (deamination) represents the conversion of the arginine into citrulline, the post-translational process taking place in a protein. This process plays a role in the formation of rigid structures (e.g., hair, skin) and is also a member of the subcortical maternal complex [46]. Recessive variants in *PADI6* genes have been associated with primary female infertility, with an early arrest during embryonic stages after ART (Assisted Reproductive Technology) and with a hydatidiform mole [46,47,48]. Missense variants and protein-truncating variants have been described, the first with milder effects than the last. Missense variants allow the stopping of the evolution after blastocyte implantation and differentiation [46].

The coexistence of molar changes with an apparently unaffected fetus is an atypical phenomenon within the familial recurrent hydatidiform mole cases. Overall, the maternal and fetal prognosis, in molar pregnancies accompanied by a viable fetus, is unfavorable [5].

Nguen and Slim proposed an algorithm for DNA testing and the subsequent genetic counselling of patients with recurrent HM. Patients with at least two HM should be tested first for mutations in the *NLRP7* gene. If the testing is positive for biallelic mutations, genetic advice is given as follows: 7% may have a normal live birth (LB), while another possibility is ovum donation. If the *NLRP7* mutation analysis is negative, the next step is *KHDC3L* testing. If the test is positive, the genetic counseling is the same. If the *KHDC3L* testing is negative, it is recommended to review the histopathology and to establish their parental distribution. For the cases with two CHM biparental diploids, the genetic counselling is the same as in the previous cases. For cases with androgenetic diploids or dispermic triploids, the chances of getting an LB from one’s own eggs are high, and ART increases the chances of an LB [31].

### 3.2. Risk Factors for Hydatidiform Mole and Progression to GTN

#### 3.2.1. Risk Factors for Hydatidiform Mole

Several risk factors have been implicated in elevating the prevalence of molar pregnancies. The risk factors are represented by age, ethnicity, antecedent molar pregnancies and family history [4]. Among the extensively researched factors, the extremes of maternal age are the most well-known factor, particularly maternal age below 20 years and over 35 years old; the latter confers a risk escalation ranging from fivefold to tenfold [2,6,8]. Triploidy resulting from fertilization between a diploid sperm (generated by diandry) and a normal egg is more common in young patients [2].

The risk profile of a complete hydatidiform mole exhibits notable variations in response to age changes. In contrast, the risk associated with a partial hydatidiform mole appears to display relatively minor fluctuations in conjunction with age, while it does not exhibit heightened occurrence among adolescents [2,7,36]. These observations are in concordance with the hypothesis that the process leading to molar pregnancy, stemming from fertilization of an aberrant oocyte, is more prone to manifest at the extremes of reproductive age. This could be attributed to higher chances of ovulating an empty ovum due to reproductive immaturity or senescence, as opposed to the probability of abnormal fertilization by two sperms [36]. While there are well-documented associations between chromosome anomalies and advanced maternal age, instances of chromosome defects in oocytes of adolescents have not been identified [7].

Additionally, antecedent molar pregnancies contribute to an increased risk of 1 to 2% for future pregnancies [6,8]. The likelihood of recurrence is on the rise with every previous molar gestation and reaches an apex in the second year after the initial event [10]. The probability of encountering a third molar pregnancy experiences a notable surge to approximately 15–20%, remaining unaffected by changes in partners, and possibly retaining a connection to either familial or sporadic instances of biparental molar disease [2]. It should be noted that patients with a partial hydatidiform mole are more likely to develop another one, as opposed to patients with a complete hydatidiform mole [10]. A woman with a previous pregnancy with a hydatidiform mole will have a 10–20% risk of having a pregnancy loss from a non-molar cause [32].

The impact of race/ethnicity on the development of molar pregnancies has also been subject to investigation. Multiple studies indicated that Asian women manifest the most pronounced susceptibility for complete moles, being over twice as likely as the white population to develop them, while concurrently displaying a diminished propensity for the development of partial moles. Furthermore, a diminished proclivity for partial mole occurrence was noted among Hispanic and black women in relation to white women [37].

Further contributing to the risk profile are factors encompassing a medical history characterized by previous instances of spontaneous abortions or infertility, dietary considerations (encompassing deficiencies in carotene and animal fats), smoking, blood group B, paternal age, maternal genetic anomalies and oral contraceptives [2,8,10]. Deficiencies in vitamins A and B9 during the first gestational weeks could deteriorate the oocytes’ differentiation in female fetuses. The insufficiency of vitamin A leads to an incomplete development of oocytes and prevents the accurate progression of meiosis II. Folate deficiencies disturb the normal proteins and DNA synthesis, which have an impact on the differentiation of the oocyte and zygote, and, additionally, they influence the instability of maternal-origin chromosomes. Both vitamin A and folates also intervene in the DNA methylation process. These methylation irregularities impact the imprinted genes within the oocyte, where the maternal methylation vanishes and is substituted by the paternal one. This phenomenon explains why affected women may develop an HM with different partners [2,49].

#### 3.2.2. Risk of GTN Progression

The placenta has its origin in the two layers of the blastocyst (cytotrophoblast and syncytiotrophoblast) associated with extra-embryonic mesoderms [2]. The hydatidiform mole is a particular condition because it originates in gestational tissue rather than maternal tissue [8]. The hydatidiform mole is in most cases benign but can become malignant and invasive [4,8]. The risk of GTN progression is different in the two forms of hydatidiform moles: 15–20% for CHMs and 0.5–1% for PHMs [25,50]. Several risk factors have been identified in correlation with the increased risk of GTN: elevated levels of hCG (over 100,000 mIU/mL), a uterus size larger than normal for gestational age and large cysts of theca lutein. Maternal age has been associated with a 3-fold risk of GTN if exceeding 50 years [51].

Since it is known that microRNA molecules are involved in various stages in the pathogenesis of cancers, the question naturally arises whether there are microRNAs involved in the progression to GTN, a hypothesis that has been the subject of several studies. A microRNA profile associated with the risk of progression to GTN was involved in complete hydatidiform moles [51].

One such factor is represented by the members of the miR-181 family. It appears that they are well expressed in the moles that will become complicated with GTN. Their function is to inhibit the expression of Apoptosis regulator Bet-2. This protein (encoded by *BCL2* genes) plays a role in suppressing apoptosis, it regulates cell death and it acts as an inhibitor of autophagy [27,28]. In the complete moles that progress to GTN, these proteins have very low levels [51].

Zhao et al. analyzed a number of microRNA molecules and identified a profile of six microRNAs with varying expression in GTN and hydatidiform moles that do not progress to GTN: miR-370-3p, miR-371a-5p, miR-518a-3p, miR-519d-3p, miR-520a3p and miR-934. The authors showed the involvement of miR-371a-5p and miR-518a-3p in the proliferation, the migration and the invasion of choriocarcinoma cells. miR-371a-5p correlated negatively with proteins encoded by the following genes: *BCCIP* (BRCA2 and CDKN1A interacting protein), *SOX2* (SRY-box transcription factor 2) and *BNIP3L* (BCL2 interacting protein 3 like). miR-518a-3p negatively correlated with proteins encoded by *MST1* (macrophage stimulating 1) and *EFNA4* (ephrin A4) [52].

The strengths of the review are the inclusion of information about various characteristics of the disease, not just the genetic part, and the highlighting of the mechanisms in correlation with the risk factors for hydatidiform moles and progression to GTN. Potential limitations of this review could be the lack of articles relevant to the topic but not identified by the search strategy. Another limitation is the paucity of studies focused on cytogenetics or molecular genetics of the hydatidiform mole.

## 4. Conclusions

Although it is a rare pathology, the hydatidiform mole is important due to its genetic heterogeneity. In this context, for proper subsequent management, it is desirable to establish the type of mole and the mechanism of its occurrence. Hydatidiform moles can be diagnosed using histopathological criteria, but in the cases of early pregnancy/borderline/difficult cases, cytogenetic techniques and, more recently, molecular biology techniques play a special role in diagnosis. The correct management is desirable to avoid the situation in which treatment is not given/an insufficient treatment is given in those cases that have a high malignant potential. Also, this approach avoids cases in which an unjustified excess treatment is given for low-risk lesions. The gestational history in conjunction with the diagnosis of the type of mole can direct the management towards testing for mutations in *NLRP7* or *KHDC3L* genes. Patients who had androgenetic and triploid dispermic moles could benefit from in vitro fertilization and preimplantation genetic screening.

The identification and knowledge of mutational mechanisms (chromosomic/genic), as well as the extension of research on the involvement of epigenetic factors in the pathogenesis of hydatidiform moles, is a prerequisite for validating diagnostic and prognostic biomarkers in various types of HM. This way, it will be possible to apply a personalized treatment associated with general management, a part of personalized medicine.

## Figures and Tables

**Table 1:** Differences between CHMs and PHMs [8,9,10,11].

Characteristics	CHM	PHM
**Histological findings**	- Abnormal chorionic villi (enlarged, irregular, polyploid or lobular aspect) which form the “cisterns” with stromal fluid trophoblastic inclusion. - Cytological atypia. - Apoptotic bodies. - Fetal stromal blood vessels are absent.	- Some of the villi are large and hydropic and others are small and fibrotic; the edges of the villi are irregular. - Small trophoblastic inclusions. - An irregular maze-like appearance given with a sketch of the formation of the central cistern is noticeable.
**Immunohistochemical findings**	Absence of p57 expression	Presence of p57 expression
**hCG**	Very high levels (>100,000)	Normal range/lower
**Uterine size**	Larger related to the expected gestational date of the pregnancy	Smaller than the suggested date
**Fetal parts**	Lack of embryonic or fetal structures	Fetal structures present
**Recurrence**	1 in 100	Small
**Risk of choriocarcinoma**	3%	Low

**Table 2:** Genes involved in recurrent hydatidiform mole [20,27,28,29].

No	Approved/Alias (Previous), Gene Symbol	Approved Gene Name	Location	Approved Protein Name	Gene/Locus MIM Number	Gene Frequency
1	***NLRP7***/ *PYPAF3*, *NOD12*, *PAN7*, *CLR19.4* (*NALP7*)	NLR family pyrin domain containing 7	19q13.42	NACHT, LRR and PYD domains-containing protein 7	609661	55%
2	***KHDC3L***/ *ECAT1* (*C6orf221*)	KH domain containing 3 like, subcortical maternal complex member	6q13	KH domain-containing protein 3	611687	5%
3	***PADI6***	Peptidyl arginine deiminase 6	1p36.13	Protein-arginine deiminase type-6	610363	1%
4	***NLRP5***/ *PYPAF8*, *MATER*. *PAN11*, *CLR19.8* (*NALP5*)	NLR family pyrin domain containing 5	19q13.43	NACHT, LRR and PYD domains-containing protein 5	609658	0.5%
5	***MEI1***/*MGC4004,2* *SPATA38*	meiotic double-stranded break formation protein 1	22q13.2	Meiosis inhibitor protein 1	608797	0.5%
6	***TOP6BL***/*FLJ22531*, *TOPOVIBL* (*C11orf80*)	TOP6B like initiator of meiotic double strand breaks	11q13.2	Type 2 DNA topoisomerase 6 subunit B-like	616109	0.5%
7	***REC114***/*LOC283677*, *FLJ27520*, *FLJ36860*, *FLJ44083*, *CT147* (*C15orf60*)	REC114 meiotic recombination protein	15q24.1	Meiotic recombination protein REC114	618421	0.5%

## Data Availability

Not applicable.
